# An Unexpected Tumor Reduction: Treatment with Olaparib Monotherapy in Heavily Pretreated BRCA2 Mutated Metastatic Pancreatic Cancer

**DOI:** 10.3390/curroncol29020049

**Published:** 2022-01-27

**Authors:** Alessandra Anna Prete, Letizia Procaccio, Francesca Bergamo, Cosimo Rasola, Floriana Nappo, Vittorina Zagonel, Sara Lonardi

**Affiliations:** 1 Oncology Unit 1, Veneto Institute of Oncology IOV–IRCCS, 35128 Padua, Italy; letizia.procaccio@iov.veneto.it (L.P.); francesca.bergamo@iov.veneto.it (F.B.); cosimo.rasola@iov.veneto.it (C.R.); floriana.nappo@iov.veneto.it (F.N.); vittorina.zagonel@iov.veneto.it (V.Z.); 2 Department of Surgery, Oncology and Gastroenterology, University of Padua, 35128 Padua, Italy; 3 Oncology Unit 3, Veneto Institute of Oncology IOV–IRCCS, 35128 Padua, Italy

**Keywords:** olaparib, metastatic pancreatic cancer, BRCA, PARP inhibitors

## Abstract

PARP inhibitors are largely recognized as active drugs in BRCA-mutated breast and ovarian malignancies. In pancreatic ductal adenocarcinoma, the PARP inhibitor olaparib has recently been approved as maintenance treatment in patients with germline BRCA mutations reaching disease control after a platinum-based first line chemotherapy, proving significant benefit on progression free survival. On the other hand, little evidence is available regarding olaparib as single agent after progression with standard treatment in BRCA-mutated pancreatic ductal adenocarcinoma. A 61-year-old female patient harboring germline BRCA2 mutation was treated at our institution for a pancreatic ductal adenocarcinoma with lung and liver metastases. The patient received three previous lines of treatment with standard therapies, as follows: after the third line treatment failure, we started a further line of treatment with olaparib in off-label prescription. After the first two cycles, a CT scan documented partial response, with complete regression of lung metastases. The response was maintained after four cycles, with further response and clinical benefit. The radiologic and clinical response was maintained for 6 months. This case highlights the potential of olaparib as single agent after progression with standard treatment in BRCA-mutated pancreatic cancer.

## 1. Introduction

Pancreatic ductal adenocarcinoma (PDAC) is an aggressive disease bearing negative prognosis: the 5-year overall survival (OS) rate does not exceed 10% and has remained unchanged for about 50 years [1,2].

Molecular DNA damage response (DDR) deficiency is one of the pathogenetic mechanisms underlying PDAC development. Breast-related cancer antigens (BRCA) mutations represent the most frequent genetic alteration leading to DDR deficiency in PDAC [3]; in detail, tumors with mutations in BRCA 1 and 2 display compromised homologous recombination (HR), resulting in inability to repair double-stranded breaks. Poly (ADP-ribose) polymerase (PARP) inhibitors are particularly effective against tumors displaying DDR; these drugs work by inhibiting the mechanism of base excision repair, leading to double-stranded breaks, which cannot be repaired due to deficient HR [4,5]. This damage is known as synthetic lethality and leads to complete inability to repair breaks on DNA with consequent cell death. PARP inhibitors have been successfully employed in the treatment of BRCA-mutated breast and ovarian malignancies and have already been approved for these tumors by the US Food and Drug Administration (FDA) and the European Medicines Agency (EMA) [6,7,8]. In PDAC, olaparib has recently been tested in a double-blind, placebo-controlled phase III trial as maintenance treatment, displaying significant benefit on progression free survival (PFS) [9], but not in OS [10]. On the other hand, no robust data exist about olaparib as single agent therapy after disease progression with standard treatment in PDAC.

Here, we describe a case of a remarkable response to olaparib single agent therapy in a heavily pretreated patient with mPDAC harboring a germline BRCA2 mutation.

## 2. Case Description

A 61-year-old female patient harboring germline BRCA2 mutation was treated at our institution for PDAC with multiple metastases.

She had been diagnosed in 2002 with a luminal B locally advanced breast cancer, treated with radical surgery and adjuvant radiotherapy, chemotherapy and hormonal therapy. In 2015, a local relapse of breast cancer occurred. She underwent mastectomy and subsequent chemotherapy and radiotherapy.

The patient referred to the following familial history of oncologic disease: her sister died from breast cancer, while her brother died from PDAC. No other significant non-oncologic diseases were referred.

On 7 August 2017, during follow up for the previous breast cancer, a pancreatic mass measuring 37 × 24 mm was observed at a CT scan—a biopsy documented PDAC. No other suspect lesions were described, and in particular no pulmonary nodules were detected.

The patient underwent surgical duodenocephalopancreasectomy on 25 September 2017. The histological examination confirmed the presence of scarcely differentiated PDAC pT2 N2 stage III per TNM staging 8th edition [11]. After surgery, the patient developed a biliopancreatic fistula, which prolonged the hospitalization.

After being discharged from hospital, on November 2017, the patient underwent a postoperative restaging CT scan which revealed two new lesions in the inferior and superior lobe of the right lung, measuring 17 and 5 mm, respectively—a biopsy confirmed pancreatic origin. In December 2017, the patient started gemcitabine and nab-paclitaxel as first line chemotherapy, achieving partial response on lung metastases per RECIST 1.1 criteria [12]. Meanwhile, the patient underwent a BRCA test, which documented a germline mutation of BRCA2; microsatellite status was also analyzed, confirming a microsatellite stable tumor (MSS).

In June 2018, pulmonary progression was observed and peripancreatic neoplastic tissue were evident at CT scan.

In July 2018, the patient started chemotherapy with FOLFOX as second line treatment reaching disease control, with stable disease per RECIST 1.1 [12] as best response.

In November 2018, pulmonary and locoregional progression was documented, so the patient started FOLFIRI as third line treatment.

**Figure 1:**
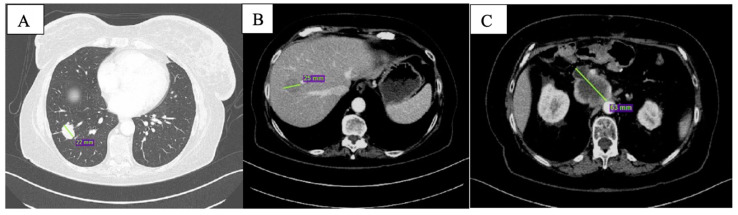
Baseline CT scan showing nodular lesion in inferior lobe of the right lung (**A**), hepatic lesion in the 8th segment (**B**) and peripancreatic tissue (**C**).

At CT scan in January 2019, both pulmonary and nodal lesions were augmented in volume; new hepatic lesions were also documented (Figure 1).

When the CT scan documented progression after the third line treatment, the patient was in good clinical condition, with an ECOG (Eastern Cooperative Oncology Group) performance status of zero. She did not complain of residual toxicities from previous treatments. Furthermore, the patient was strongly motivated to continue treatment; she also had optimal support from caregivers. The biochemistry and blood examinations were in range. No jaundice was documented. Considering the permissive clinical conditions confirmed by laboratory tests, the strong willingness to continue therapy and the known BRCA mutation, we decided to start a further line of treatment with olaparib in off-label prescription. The patient accepted the treatment and started the first cycle in February 2019 with a dosage of olaparib 400 mg bid capsules.

**Figure 2:**
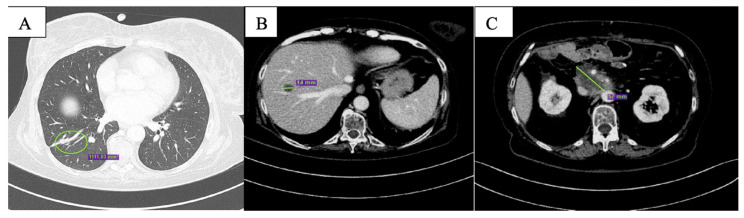
CT scan after the first 2 months of olaparib showing complete response of the nodular lesion in inferior lobe of the right lung (**A**), reduction in size of the hepatic lesion in the 8th segment (**B**) and of peripancreatic tissue (**C**).

After the first two months of therapy, the patient displayed complete response on the lung metastases (Figure 2A); overall, partial response was documented (Figure 2B,C). One single new liver lesion of uncertain origin was documented. After staff consultation, considering the good clinical conditions, the absence of toxicities from treatment, except for G1 nausea per CTCAE v. 5.0 [13], the diameter of the new lesion (<10 mm), and the excellent response obtained on the other lesions, we decided to continue with the same therapy.

**Figure 3:**
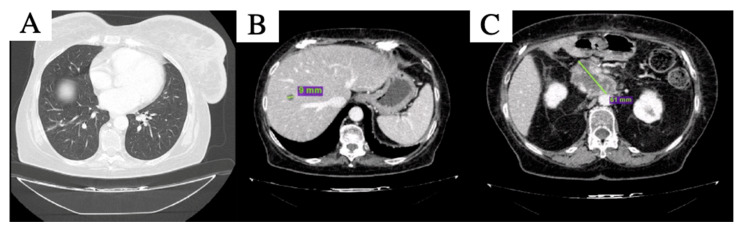
CT scan after 4 months of olaparib: complete disappearance of the nodular lesioni in inferior lobe of the right lung was confirmed (**A**), the hepatic lesion in the 8th segment (**B**) is further reduced in size and peripancreatic tissue is slightly increased and substancial stability of the peripancreatic tissue was described (**C**).

After 4 months of therapy, the new liver lesion was no longer visible; of the other two remaining liver lesions, one reached complete response and the other was further reduced in maximum diameter, while the peripancreatic tissue was stable (Figure 3). Pulmonary lesions were still undetectable, maintaining complete response. Thus, partial response per RECIST 1.1 [12] was declared. The treatment was well tolerated, except for G3 anemia per CTCAE v. 5.0 [13], which required blood transfusions and one level reduction in olaparib dosage after the third cycle, i.e., 200 mg bid capsule. Clinical benefit was also observed, with improved self-care and pain control.

**Figure 4:**
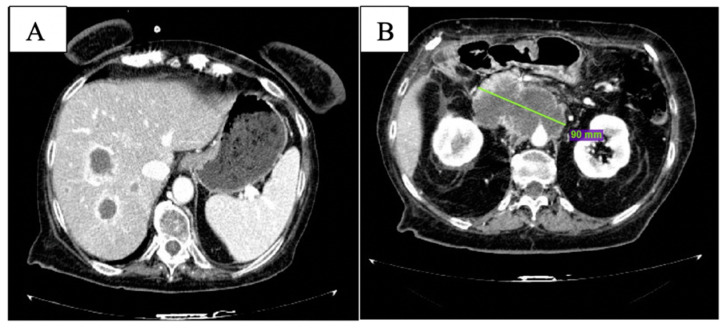
CT scan after 6 months of olaparib: new liver lesions are documented (**A**) and peripancreatic tissue is increased (**B**).

After the 6th month of therapy with olaparib, clinical conditions worsened with uncontrolled abdominal pain and G3 fatigue per CTCAE v. 5.0 [13]. At CT scan, multiple new liver lesions were observed (Figure 4A); the peripancreatic tissue was augmented with celiac trunk encasement (Figure 4B). Peritoneal carcinosis was also evident.

The treatment was stopped in July 2019, and the patient died on 9 August 2019.

## 3. Discussion

At present, few effective treatments are available for metastatic PDAC; furthermore, this malignancy is often refractory to anticancer drugs. PDAC is characterized by extremely elevated intertumoral heterogeneity [14]; as a result, finding druggable targets is particularly challenging and molecular pathology of PDAC is currently under study with the aim of identifying novel driver mutations. The case we presented is a rare example of precision medicine applied to PDAC. Two elements were of crucial importance, as follows: having tested the patient for BRCA mutation and the pre-existing evidence about the efficacy of PARP inhibitors in BRCA-mutated malignancies.

Germline BRCA mutations are found in a small subgroup of patients, accounting for no more than 4% of PDAC [15]. The prognostic role of BRCA mutations in ovarian and breast cancer is well established; BRCA2 mutated breast cancer displays worse prognosis compared to sporadic cancer, while, in BRCA1, no substantial differences were observed [16]. In ovarian cancer, BRCA mutations have been associated with better prognosis [17]. In PDAC, it is hard to establish a correlation between BRCA mutations and survival due to the rarity of these cases; however, in a retrospective analysis conducted on a large cohort of BRCA-mutated PDAC, Golan et al. described a slight increase in OS compared to historical cohorts of non-BRCA-mutated PDAC [18].

Despite their low incidence in PDAC, detecting BRCA mutations is becoming a matter of growing interest, both to individuate hereditary predisposition in healthy relatives and for potential therapeutic and prognostic implications in patients affected. In Italy, specific recommendations have been recently released establishing the indication to the test for germinal BRCA mutations for all patients aged less than 75 years who are affected by PDAC [19]. As already said, a growing body of evidence suggests that tumors characterized by DDR, such as BRCA-mutated malignancies, are particularly sensitive to therapeutic agents with synthetic lethality. A class of anticancer drugs characterized by synthetic lethality is that of platinum salts. These agents show high activity in BRCA-mutated breast and ovarian cancer [20,21], and some evidence suggests the effectiveness of platinum salts in PDAC [22]. PARP inhibitors represent a more recent class of agents with synthetical lethality. Olaparib has recently been tested as maintenance therapy after first line treatment in metastatic PDAC in the POLO trial, as follows: eligible patients were diagnosed with metastatic PDAC (mPDAC), harboring germline BRCA1 or 2 mutation and had to have achieved disease control after platinum-based first line chemotherapy. The study met its primary endpoint: progression free survival (PFS) reached 7.4 months in the treatment group vs. 3.8 months in the placebo group, with a hazard ratio (HR) for disease progression or death of 0.53 (95% confidence interval, CI 0.35 to 0.82) and a *p*-value of 0.004 [9]. Unfortunately, both initial interim [9] and updated [10] analysis did not show significant advantage of olaparib as maintenance therapy on OS, although a trend toward better OS in patients treated with olaparib was documented.

After the POLO trial, other studies addressed the employment of PARP inhibitors in PDAC. O’Reilly and colleagues conducted a phase II trial of gemcitabine and cisplatin with or without veliparib in first line for PDAC with BRCA/PALB2 mutations, with response rate (RR) as primary endpoint. The study did not reach its primary endpoint; despite this, OS results exceeded the pre-study expectations. Furthermore, in an exploratory cohort, veliparib as maintenance treatment was investigated; median OS (mOS) reached 23.4 months, performing even better than olaparib in the POLO trial [23].

In our case, olaparib was used as monotherapy after failure of standard chemotherapeutic treatments, giving remarkable clinical and radiological response. Nonetheless, few are the studies supporting the employment of olaparib in such setting in metastatic PDAC. In a phase II study, Kaufman et al. described the activity of olaparib in terms of tumor response rate in a cohort of pretreated patients diagnosed with different tumor types and with germline BRCA1/2 mutations. In total, 23 patients with mPDAC were enrolled; in this small cohort, a tumor response rate of 21.7% was observed [24]. Given these encouraging results and the few effective antineoplastic treatments currently available for PDAC, it would be interesting to explore PARP inhibitors in settings different from maintenance after first line. Rucaparib was also tested as single agent in a phase II trial in patients with pretreated, BRCA-mutated, metastatic PDAC, showing promising activity [25].

Of note, about 24% of PDAC show genetic alterations which lead to DDR. Among these are PDAC subtypes with molecular, histological and clinical features similar to those displayed by BRCA-mutated tumors in absence of detectable BRCA mutations; this phenotype is known as BRCAness [26,27]. Testing PARP inhibitors in these patients would be of crucial importance to extend this intriguing therapeutic opportunity to a larger group of patients. Previous examples of therapy with PARP inhibitors in non-BRCA-mutated patients came from studies on ovarian cancer. In the recently published ENGOT-OV16/NOVA trial, patients affected by ovarian, fallopian tube, or peritoneal cancer in response after platinum rechallenge were enrolled into two separate cohorts based on presence or absence of detectable BRCA mutations. Patients in both cohorts were randomized to receive niraparib 300 mg once daily or a placebo. Statistically significant benefit in PFS was observed in both BRCA-mutated and non-BRCA-mutated cohorts [28].

A concern of BRCA-mutated tumors is their ability to develop resistance against agents with synthetic lethality; several studies have demonstrated that BRCA-mutated tumors can restore the HR mechanism during therapy with platinum agents or PARP inhibitors. The most accepted theory is that the development of secondary BRCA mutations would reactivate the HR mechanism, thus rendering the tumor refractory to agents with synthetical lethality. The studies conducted to test this hypothesis showed different BRCA mutations in tissue from primary tumor and in tissue from metastases; as a consequence, identifying the BRCA mutation profile could be useful to predict response to platinum agents or to PARP inhibitors [29].

Another therapeutic strategy deserving special mention is immunotherapy; recently, Marabelle et al. published the results of the phase II KEYNOTE-158 trial in which the immune checkpoint inhibitor (ICI) pembrolizumab was tested in gastrointestinal, non-colorectal neoplasms in advanced stage and characterized by high microsatellite instability (MSI-H) or deficiency in DNA mismatch repair (dMMR) and progressed after prior therapy; primary endpoint was objective response rate (ORR). In this trial, 22 (9.4% of the total) patients affected by PDAC were enrolled; among them, ORR was 18.2% (95% CI 5.2 to 40.3), while PFS and OS in moths were 2.1 (95% CI 1.9 to 3.4) and 4.0 (95% CI 2.1 to 9.8), respectively [30]. Based on these results, the FDA approved pembrolizumab for metastatic MSI-H/dMMR PDAC progressed after previous treatments. Furthermore, moving from such encouraging results from KEYNOTE-158, several studies are currently ongoing to better understand possible employments of ICI in PDAC [31]. Intriguingly, a recent publication from Zhou et al. [32] suggest that in metastatic PDAC, high TMB and BRCA2 alteration could represent a potential biomarker predictive of response to ICIs. This observation clearly deserves further studies to be confirmed, but it could represent one more important step toward personalized treatment in PDAC.

## 4. Conclusions

We presented a case of a remarkable clinical and radiological response to olaparib in a heavily pretreated patient with BRCA2 germline mutation. This case underlines the importance of investigating therapy with PARP inhibitors after failure of standard therapies and of testing for BRCA in patients with suggestive personal and familial history.
